# Early detection and tracking of bulbar changes in ALS via frequent and remote speech analysis

**DOI:** 10.1038/s41746-020-00335-x

**Published:** 2020-10-13

**Authors:** Gabriela M. Stegmann, Shira Hahn, Julie Liss, Jeremy Shefner, Seward Rutkove, Kerisa Shelton, Cayla Jessica Duncan, Visar Berisha

**Affiliations:** 1 Arizona State University, Phoenix, AZ USA; 2 Aural Analytics, Scottsdale, AZ USA; 3 Barrow Neurological Institute, Phoenix, AZ USA; 4 Beth Israel Deaconess Medical Center, Harvard Medical School, Boston, MA USA

**Keywords:** Amyotrophic lateral sclerosis, Predictive markers

## Abstract

Bulbar deterioration in amyotrophic lateral sclerosis (ALS) is a devastating characteristic that impairs patients’ ability to communicate, and is linked to shorter survival. The existing clinical instruments for assessing bulbar function lack sensitivity to early changes. In this paper, using a cohort of *N* = 65 ALS patients who provided regular speech samples for 3–9 months, we demonstrated that it is possible to remotely detect early speech changes and track speech progression in ALS via automated algorithmic assessment of speech collected digitally.

## Introduction

Amyotrophic lateral sclerosis (ALS) is characterized by a progressive loss of motor function due to central nervous system damage and loss of spinal and bulbar motor neurons. ALS causes individuals to become progressively weaker and lose motor function, eventually resulting in death. Social and economic consequences of ALS include cost of care for the patients, loss of employment, and cost of treatment, medications, and orthopedic devices^1–3^. Bulbar deterioration is particularly devastating, impairing the ability to communicate, leading to faster decline, shorter survival (less than 2 years from diagnosis), and reduced quality of life^4–6^. Studies have found that while 30% of individuals in the population present with bulbar symptoms at the onset of ALS, most ALS patients eventually develop them and lose their ability to speak and swallow safely^7^.

The standard ways of assessing bulbar dysfunction are the ALS functional rating scale-revised (ALSFRS-R) and, less commonly, the Center for Neurologic Study Bulbar Function Scale (CNS-BFS)^8^. Both instruments, however, lack sensitivity to early bulbar changes^9^. Several studies have found that speech features, such as jitter, shimmer, articulatory rate, speaking rate, and pause rate, are affected in ALS^10,11^, and that these can be measured from remotely-collected speech samples^12,13^. However, no study has assessed the sensitivity of remote speech analysis in detecting and tracking bulbar change. In this study, we assessed speech features digitally and evaluated their sensitivity to detecting early changes and tracking progression.

We defined early changes as speech changes that occurred before any changes in the ALSFRS-R bulbar subscales. We defined sensitive tracking as the ability to detect longitudinal within-person changes in speech. We used a cohort of healthy and ALS patients from ALS at Home^14^, a longitudinal, observational study that was conducted entirely remotely. Participants were recruited, screened, enrolled, and assessed daily from home. Speech was collected via a mobile application and assessed through automated speech analysis. Although it is possible to analyze a large number of speech features, we focused on articulatory precision (AP) and speaking rate (SR) as they relate to articulation and rate, both of which are known to decline in dysarthria^15^ secondary to ALS. We evaluated whether the automatic analysis of remotely-collected speech could (1) detect early speech changes and (2) sensitively track speech changes longitudinally.

The ALS sample was divided according to the following categories:

- *Impairment* category: We identified participants who had normal function according to ALSFRS-R bulbar subscales (speech, salivation, and swallowing subscales with score = 4) at the beginning of the study. Twelve participants had *normal bulbar function* and the other participants had *impaired bulbar function*. This sample was used to test whether AP and SR significantly differed between the *normal bulbar function* group and the *healthy* controls, thus evaluating their ability to detect early changes.
- *Onset* category: Type of onset was collected from participants. Twelve ALS participants initially presented with *bulbar onset*, while the other 52 participants presented with other types of onset (*nonbulbar onset*). The non-bulbar onset group included participants with axial, limb, and generalized onset. This sample was used to compare the SR and AP longitudinal trajectories of individuals according to their type of onset (bulbar and nonbulbar onset). We expected that bulbar-onset participants would exhibit faster speech decline, and thus used onset type to evaluate whether AP and SR were sensitive to these differences in speech decline.

## Results

### Description of sample

**Fig. 1:**
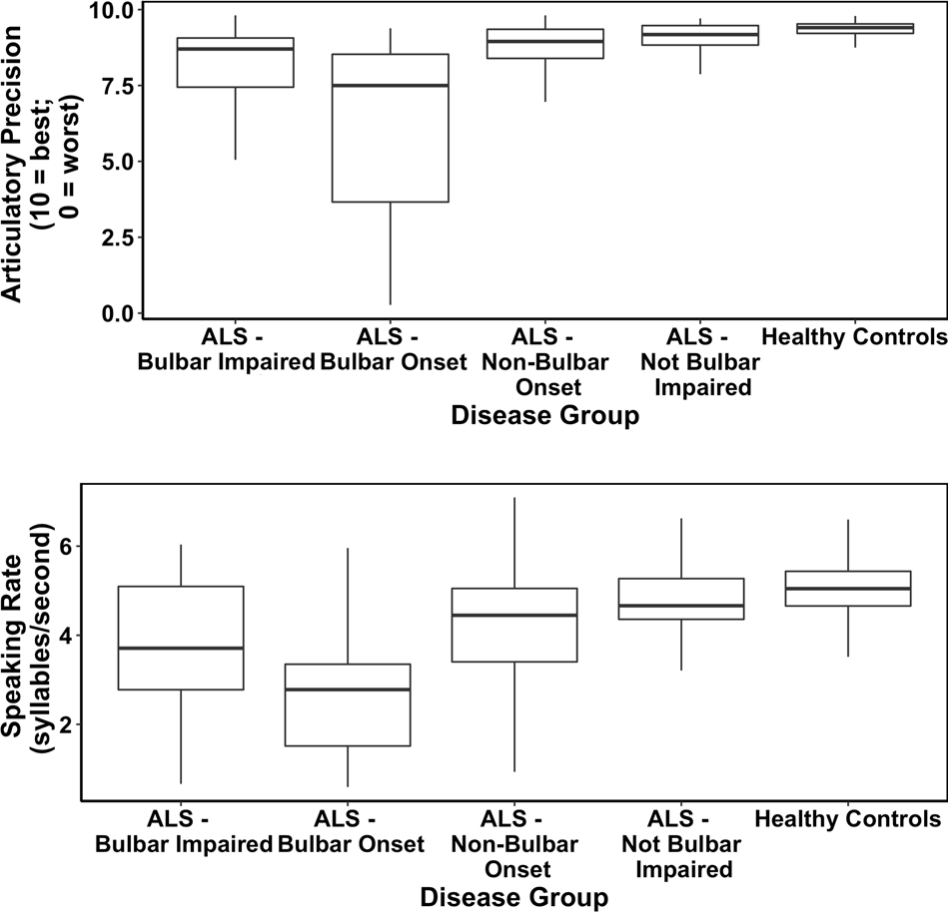
Boxplots for healthy controls, ALS participants with no bulbar impairment (normal ALSFRS-R scores for speech, swallowing, and salivation), ALS participants with bulbar impairment (at least one score below 4 in the ALSFRS-R scores for speech, swallowing, and salivation), and ALS participants with bulbar-onset (type of ALS onset). All data points are used. Note: the mixed-effects models indicated that the healthy controls significantly differed from all ALS participants (*p*-value = 0.005 and *p*-value < 0.001 for AP and SR, respectively) and from ALS participants with no bulbar impairment (*p*-value = 0.024 and 0.009 for AP and SR, respectively). Center line = median; box limits = upper and lower quartiles; whiskers = 1.5× interquartile range.

Tables 1 and 2 show the descriptive statistics of the sample, including their demographics and ALS severity. The ALSFRS-R speech, ALSFRS-R bulbar, SR, and AP scores all indicate that the most severe group in terms of bulbar symptoms were the ALS participants with bulbar onset, followed by ALS participants with bulbar impairment. Overall, lower scores in AP and SR were associated with greater impairment in speech (mixed-effects^16^ correlations between the ALSFRS-R speech subscale and AP, SR were *r* = 0.73, *r* = 0.64, respectively; Lorah^16^). Figure 1 shows the distributions of the AP and SR scores for healthy, ALS with normal bulbar function, impaired bulbar function, bulbar onset, and nonbulbar onset participants.

**Table 1:** Sample description (enrollment).

Total number of participants	86
Total number of observations	8416
Number of healthy; ALS	21; 65
ALS participants: number of participants with normal bulbar function	11
ALS participants: number of participants with bulbar onset	12
Average length of enrollment	203.9 days
Average frequency of data collection	Every 2.9 days
Gender for ALS and healthy participants (% females)	ALS 35%; healthy 71%
Mean (standard deviation) years since first symptom onset	2.9 (2.0)
Average (standard deviation) age for ALS and healthy participants	ALS 61 (10.2); healthy 55 (12.5)

**Table 2:** Sample Description (by group).

Sample description	Healthy	All ALS	ALS with Bulbar Onset	ALS with Non-Bulbar Onset	ALS with Bulbar Impairment	ALS with No Bulbar Impairment
ALSFRS-R speech mean (standard deviation); scale 0–4, higher is better	–	3.1 (1.0)	1.9 (0.89)	3.5 (0.70)	2.7 (1.0)	4 (0)
ALSFRS-R bulbar mean (standard deviation); 0–12, higher is better	–	9.7 (2.6)	6.2 (2.6)	10.7 (1.5)	8.4 (2.5)	12 (0)
ALSFRS-R total mean (standard deviation); scale 0–48, higher is better	–	37.1 (5.7)	38.0 (4.9)	36.9 (5.8)	35.9 (5.5)	41.6 (3.1)
Speaking rate mean (standard deviation); syllables/second, higher is better	5.1 (0.6)	3.9 (1.3)	2.7 (1.4)	4.2 (1.2)	3.6 (1.4)	4.6 (0.7)
Articulatory precision mean (standard deviation); scale 0–10, higher is better	9.4 (0.3)	8.2 (1.8)	6.3 (2.7)	8.6 (1.4)	7.8 (2.1)	9.0 (0.6)

### Analyses

Three sets of analyses were conducted. First, to evaluate whether declines in AP and SR occurred earlier than declines on the ALSFRS-R bulbar subscale, we compared the healthy individuals to ALS individuals with normal bulbar function. If participants started the study with normal bulbar function but their ALSFRS-R bulbar scores declined throughout the study due to ALS progression, we only used their data before the decline began. Both AP and SR were significantly higher in the healthy individuals than in the ALS individuals with normal bulbar function (see top section of Table 3), indicating that AP and SR decline was detected earlier than declines on the ALSFRS-R bulbar subscale.

**Table 3:** Results from all analyses.

Analysis	Estimate (standard error)	*p*-value^c^
Mixed-effects models for comparisons between healthy controls and ALS with normal bulbar function		
Articulatory precision	−0.62 (0.29)^a^	0.0236
Speaking rate	−0.77 (0.28)^a^	0.009
Mixed-effects models for comparisons between healthy controls and all ALS		
Articulatory precision	−0.82 (0.28)^a^	0.005
Speaking rate	−1.01 (0.25)^a^	<0.001
Growth curve model for articulatory precision		
Fixed-effects parameters		
Intercept	9.94 (0.37)	
Slope for nonbulbar-onset participants (change per month)	−0.03 (0.02)^b^	0.025
Difference in slope between bulbar and non-bulbar -onset participants	−0.12 (0.03)^b^	<0.001
Random effects parameters		
Intercept standard deviation	2.54	
Slope standard deviation	0.10	
Correlation between intercepts and slopes	−0.67	
Residual standard deviation	0.33	
Growth curve model for speaking rate		
Fixed-effects parameters		
Intercept	4.21 (0.25)	
Slope for non-bulbar -onset participants (change per month)	−0.003 (0.007)^b^	0.960
Difference in slope between bulbar and non-bulbar -onset participants	−0.05 (0.01)^b^	<0.001
Random effects parameters		
Intercept standard deviation	1.57	
Slope standard deviation	0.04	
Correlation between intercepts and slopes	−0.70	
Residual standard deviation	0.25	

^a^For group comparison analyses, the metrics were standardized to have mean 0 and standard deviation 1 for easier interpretation of the coefficients^16^.

^b^For growth curve models, the slopes are scaled to the amount of change per month. *p*-values are provided for slopes; no *p*-values are available for intercepts or random effects parameters.

^c^*p*-values calculated using *χ*^*2*^ likelihood ratio tests.

Second, we further evaluated the validity of AP and SR as a measure of speech decline in ALS by comparing the scores in healthy controls and all ALS participants regardless of bulbar impairment or onset. AP and SR were significantly higher in healthy participants than all ALS participants regardless of onset or impairment (middle section of Table 3), strengthening the evidence that these two measures can detect ALS speech impairment.

**Fig. 2:**
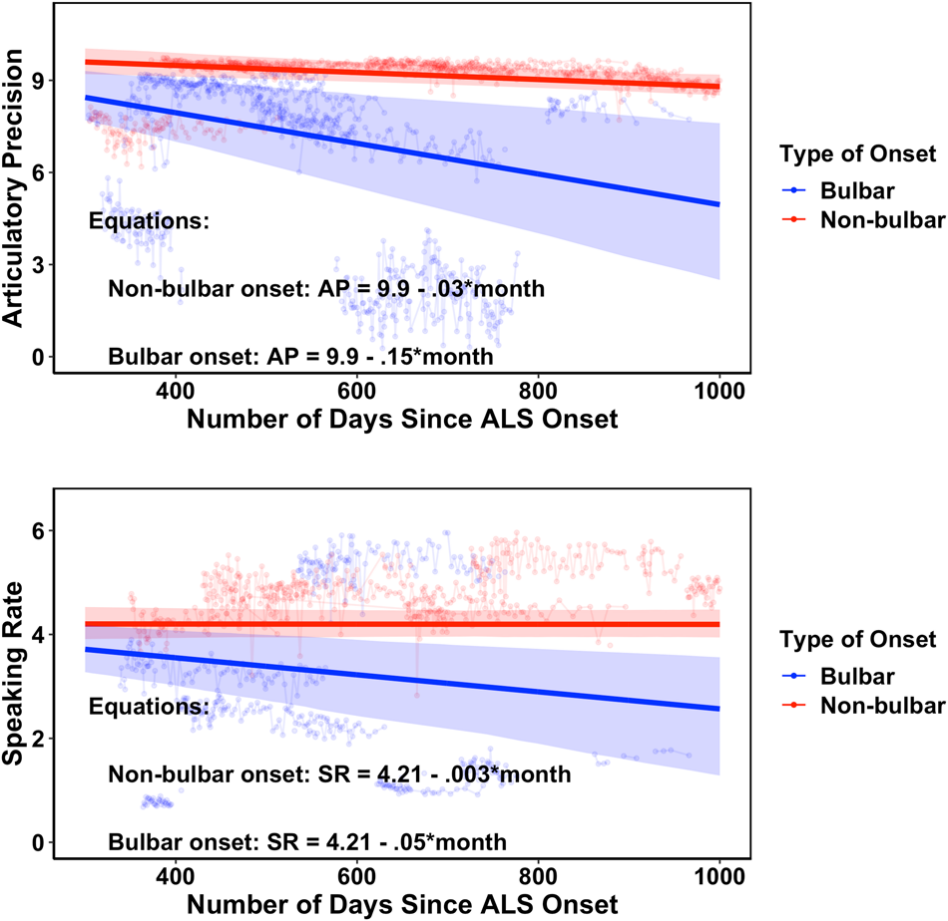
Articulatory precision (AP) and speaking rate (SR) scores as a function of number of days since date of ALS onset. AP was extracted from data collected for the participants’ full duration of enrollment, whereas for SR, the initial 45 days were excluded to avoid rate increases which were observed in healthy controls, possibly due to familiarization with the sentences. Note: The thin lines and points show the observed data of a random sample of 35 participants. The dark red thick line represents the expected trajectory for all nonbulbar participants. The dark blue thick line represents the expected trajectory for all bulbar participants. The expected trajectories were obtained from the fixed-effects estimates from the growth curve models. The shaded areas indicate the bootstrap 95% confidence bands for the expected trajectories. In the equations: 1 month = 30 days.

Third, we evaluated the sensitivity of AP and SR to detect longitudinal within-person changes in speech. We used a growth curve model^17^ (GCM), which is a mixed-effects model that estimates the longitudinal trajectory of an outcome for a sample of the participants with multiple observations over time. We compared the rates of decline between the bulbar-onset and nonbulbar-onset participants expecting that bulbar-onset participants would have steeper speech decline than nonbulbar onset participants. The time variable was the number of days since the onset of the first symptom. For both features, the final GCM^17^ followed a linear trajectory, had a random intercept and random slope, and had distinct mean slopes for bulbar-onset and nonbulbar-onset participants. For AP, both groups had significantly negative mean slopes, such that AP decreased as ALS progressed. However, bulbar-onset participants declined more rapidly as their mean slope was significantly more negative than the mean slope for nonbulbar-onset. For SR, the decline over time in nonbulbar-onset participants was nonsignificant (mean slope not significantly different from 0), whereas the bulbar-onset group showed significant decline (the mean slope was negative and significantly lower than nonbulbar-onset group). The longitudinal plots are shown in Fig. 2, and the GCM parameters are in the bottom section of Table 3.

## Discussion

In this study, we have identified two objective speech metrics that detected bulbar impairment before the ALSFRS-R bulbar subscale, sensitively tracked longitudinal decline, and could be assessed from remotely collected speech samples via a mobile app. They were consistent with both cross-sectional and longitudinal expectations: cross-sectionally, healthy participants had the highest SP and AP, followed by ALS participants with no bulbar impairment, and finally followed by all ALS participants, including those with bulbar impairment. Furthermore, the analyses were repeated controlling for time of day, age, and gender, and the results remained consistent. Longitudinally, bulbar-onset ALS participants declined faster in SR and AP than nonbulbar-onset ALS participants. This represents a unique opportunity for earlier and more sensitive identification and remote tracking of bulbar impairment than is currently available.

The ability to digitally detect early changes and sensitively track progression has important implications for personal planning and for research. Such information is valuable to the patient, family, and medical staff to inform life planning decisions, such as making necessary work and family decisions while speech is still intelligible, deciding on the timing of therapeutic interventions, and obtaining augmented and alternative communication technology^18^. These objective measures are also useful for ALS clinical trials as they can be used to provide valuable information about disease progression, determine enrollment, stratify participants, and appropriately power a study^19^. Furthermore, the ability to remotely assess participants in a study has the additional benefit of reducing participant burden, reducing attrition, and enrolling individuals who would otherwise not be able to participate, such as those with transportation or ambulation challenges.

One limitation of the study was that participant information such as cognitive function, drinking, smoking, vision problems, medications, ability to read, or other health problems was not available, and therefore we were not able to explore these as potential confounders. However, given the consistency of the results, we do not expect that controlling for these additional variables would lead to a different conclusion, although a prospective study is needed to confirm this. Other limitations of remote assessment include misperformance of tasks, for example, reading a sentence incorrectly. We screened for this by automatic QA on all samples and random manual QA on a subset of samples.

## Methods

### Sample

The study was approved by the institutional review board at Barrow Neurological Institute. All participants provided written informed consent to participate in the study. Participants from ALS at Home provided daily speech samples for 3 months, twice weekly for an additional 6 months, and ALSFRS-R scores on a weekly basis. Participants were allowed to receive assistance from their caregivers if needed. In the current analysis, we included participants who were enrolled for at least 45 days to use participants that were engaged in the study and avoid those who dropped out too early. This resulted in 21 healthy participants and 65 participants with ALS.

### Speech collection and analysis

**Fig. 3:**
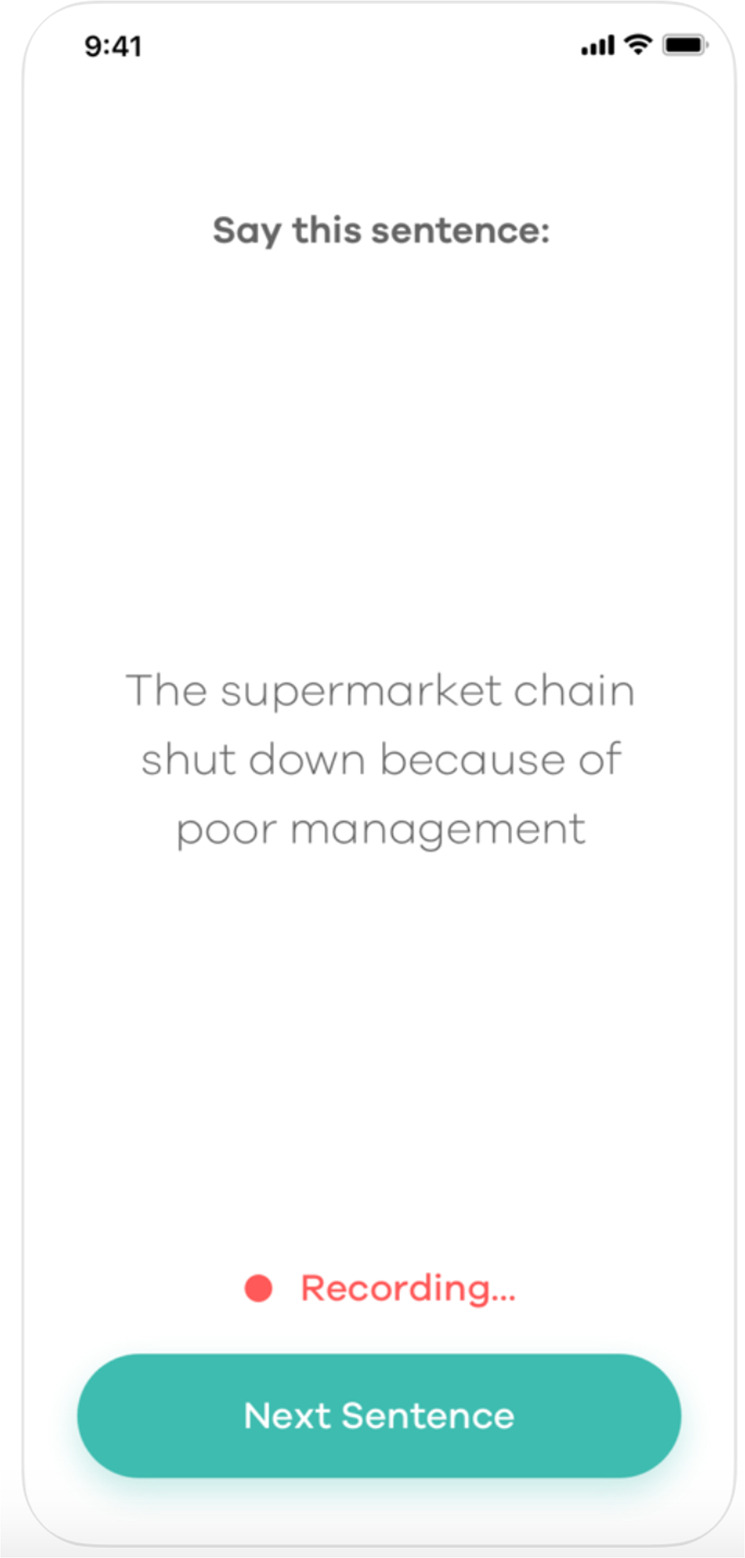
Screenshot of the mobile application. This figure illustrates a sentence from the mobile application used for collecting speech samples.

Speech samples were collected remotely via a mobile application^20^, where participants were requested to complete a series of speech elicitation tasks, including readings of five sentences. The instructions, including the sentences, were provided in the application, and participants read the text from the application. The same text was shown each day to all participants. Figure 3 shows a screenshot of the app. Speech was recorded locally on the participants’ phones, uploaded to a separate cloud-based repository, saved as a.wav file, and algorithmically analyzed on the cloud. Participants were requested to make the recordings from a quiet room, and ambient noise was recorded for 5 s and used in the speech analysis.

The speech obtained from the five sentences was used to extract SR and AP^14,20^. SR is a measure of how fast participants read the sentences. The SR is determined by automatically estimating the total speech time from the read sentences and dividing the number of syllables in the target sentences by the total speech time. To determine the speech onset and offset times, we use a statistical model-based voice activity detector similar to the one described in Sohn et al.^21^. This model uses spectral and energy features extracted from the collected background noise sample to identify an optimal speech detection threshold. The total speech time is then measured by finding the time elapsed from speech onset to speech offset. The number of syllables is known as the participant is asked to read specific sentences. The speaking rate is the total number of syllables divided by the total speech time. AP is a measure of the match between the expected and observed acoustic features for each phoneme. The algorithm, an extension of existing work^22^, takes as input connected speech, elicited from the speaker via the mobile app, and the corresponding transcript. The algorithm assesses how well the acoustics of each phoneme correspond to the acoustics of the expected phoneme in spoken English. This assessment is made by creating a distribution of acoustic features for every English phoneme from a large corpus of read speech (~1000 h) in American English. We then calculate a likelihood ratio from a comparison between the acoustic features extracted from each phoneme in the speech collected by the app and the normative distribution for the expected phoneme. For ease of interpretation, articulatory precision was projected onto a 0–10 scale (higher scores are indicative of more precise articulation).

### Statistical analyses

Given that each participant had repeated observations, the analysis necessitated mixed-effects models, where fixed-effects parameters were used for estimating the mean difference between the two groups and the mean trajectories. All analyses were performed in R. The packages lme4^23^ and nlme^24^ were used, since these two are widely used R packages to estimate mixed-effects models.

### Reporting summary

Further information on experimental design is available in the Nature Research Reporting Summary linked to this paper.

## Supplementary information

Reporting Summary

## Data Availability

The data that support the findings of this study are available from the corresponding author upon request.

## Code availability

All analyses were conducted in R language. The code is available from the corresponding author upon request.
