# Author Correction: Early detection and tracking of bulbar changes in ALS via frequent and remote speech analysis

**DOI:** 10.1038/s41746-020-00364-6

**Published:** 2020-11-20

**Authors:** Gabriela M. Stegmann, Shira Hahn, Julie Liss, Jeremy Shefner, Seward Rutkove, Kerisa Shelton, Cayla Jessica Duncan, Visar Berisha

**Affiliations:** 1 Arizona State University, Phoenix, AZ USA; 2 Aural Analytics, Scottsdale, AZ USA; 3 Barrow Neurological Institute, Phoenix, AZ USA; 4 Beth Israel Deaconess Medical Center, Harvard Medical School, Boston, MA USA

**Keywords:** Amyotrophic lateral sclerosis, Predictive markers

Correction to: *npj Digital Medicine* 10.1038/s41746-020-00335-x, published online 13 October 2020

The original version of this Article omitted the following from the Acknowledgements:

ALS Finding a Cure Grant

This has now been corrected in both the PDF and HTML versions of the Article.

