# Supplementary material for: Early detection and tracking of bulbar changes in ALS via frequent and remote speech analysis
Source: NPJ Digit Med. 2020 Oct 13;3:132. doi: 10.1038/s41746-020-00335-x (PMC7555482; doi:10.1038/s41746-020-00335-x)
Supplement: Supplementary file 1 — Reporting Summary [file 41746_2020_335_MOESM1_ESM.pdf]

nature research

Corresponding author(s): Gabriela Stegmann

Last updated by author(s): Aug 3, 2020

Reporting Summary

Nature Research wishes to improve the reproducibility of the work that we publish. This form provides structure for consistency and transparency in reporting. For further information on Nature Research policies, see our Editorial Policies and the Editorial Policy Checklist.

Statistics

For all statistical analyses, confirm that the following items are present in the figure legend, table legend, main text, or Methods section.

n/a	Confirmed	
☐	☒	The exact sample size (*n*) for each experimental group/condition, given as a discrete number and unit of measurement
☐	☒	A statement on whether measurements were taken from distinct samples or whether the same sample was measured repeatedly
☐	☒	The statistical test(s) used AND whether they are one- or two-sided<br>*Only common tests should be described solely by name; describe more complex techniques in the Methods section.*
☐	☒	A description of all covariates tested
☐	☒	A description of any assumptions or corrections, such as tests of normality and adjustment for multiple comparisons
☐	☒	A full description of the statistical parameters including central tendency (e.g. means) or other basic estimates (e.g. regression coefficient) AND variation (e.g. standard deviation) or associated estimates of uncertainty (e.g. confidence intervals)
☐	☒	For null hypothesis testing, the test statistic (e.g. *F*, *t*, *r*) with confidence intervals, effect sizes, degrees of freedom and *P* value noted<br>*Give P values as exact values whenever suitable.*
☒	☐	For Bayesian analysis, information on the choice of priors and Markov chain Monte Carlo settings
☐	☒	For hierarchical and complex designs, identification of the appropriate level for tests and full reporting of outcomes
☐	☒	Estimates of effect sizes (e.g. Cohen's *d*, Pearson's *r*), indicating how they were calculated

Our web collection on statistics for biologists contains articles on many of the points above.

Software and code

Policy information about availability of computer code

Data collection	Speech data collection was conducted through a commercial app called ALS at Home. The app can be downloaded for free from the app store (https://apps.apple.com/in/app/als-at-home-speech/id1169813257) and was created by Aural Analytics (https://auralanalytics.com/). The study uses the 2016 version, which requires iOS 8.0 or later and is compatible with iPhone, iPad and iPod touch.
Data analysis	The data analysis was conducted in R language version 3.6.1 (https://www.r-project.org/). The packages used for conducting the analyses were lme4 (2015 version; https://cran.r-project.org/web/packages/lme4/index.html) and nlme (2019 version; https://cran.r-project.org/web/packages/nlme/index.html).<br>The R code used for data analysis and data set are available upon request from the corresponding author.

For manuscripts utilizing custom algorithms or software that are central to the research but not yet described in published literature, software must be made available to editors and reviewers. We strongly encourage code deposition in a community repository (e.g. GitHub). See the Nature Research guidelines for submitting code & software for further information.

Data

Policy information about availability of data

All manuscripts must include a data availability statement. This statement should provide the following information, where applicable:

- Accession codes, unique identifiers, or web links for publicly available datasets
- A list of figures that have associated raw data
- A description of any restrictions on data availability

The data that support the findings of this study are available from the corresponding author upon request.

Field-specific reporting

Please select the one below that is the best fit for your research. If you are not sure, read the appropriate sections before making your selection.

☐ Life sciences ☒ Behavioural & social sciences ☐ Ecological, evolutionary & environmental sciences

For a reference copy of the document with all sections, see nature.com/documents/nr-reporting-summary-flat.pdf

Behavioural & social sciences study design

All studies must disclose on these points even when the disclosure is negative.

Study description	This was a longitudinal, observational study. Both qualitative (e.g., gender) and quantitative data (e.g., age, speech metrics) were collected.
Research sample	This is the same sample as the one from Rutkove SB, Qi K, Shelton K, Liss J, Berisha V, Shefner JM. ALS longitudinal studies with frequent data collection at home: study design and baseline data. Amyotrophic Lateral Sclerosis and Frontotemporal Degeneration. 2019;20(1-2):61-67. doi:10.1080/21678421.2018.1541095.<br>The name of the study was ALS at Home. This consisted of a sample of individuals with amyotrophic lateral sclerosis and healthy controls.<br>Mean age = 61 y.o. (ALS) and 55 y.o. (healthy).<br>Diagnosis distribution: 65 ALS; 21 healthy.<br>Gender distribution: ALS 35% F; healthy 71% F.
Sampling strategy	This was a non-probability sample which was collected completely remotely. Participants were recruited through the CDC ALS patient registry and the ALS Association and the Muscular Dystrophy Association websites. The study was advertised through Facebook, Google Ads, Twitter, and Reddit. ALS clinics were also used for recuiting.
Data collection	Data collection was done at home. The patients' caregivers were allowed to assist if needed. Speech data was collected using a mobile application (https://auralanalytics.com/).
Timing	Data collection was started in 2017 and ended in 2019. Participants were asked to provide speech samples on a daily basis for the first 3 months and twice per week for the following 6 months.
Data exclusions	Diagnosed and actively undergoing treatment for cancer, heart failure, end stage renal disease, or another significant medical condition deemed by the PI to likely affect the participant's ability to comply with the protocol,<br>Unwilling or unable to comply with the requirements of this protocol, including the presence of any condition (physical, mental, or social) that is likely to affect the participant's ability to comply with the protocol,<br>Any other reasons that, in the opinion of the PI, the candidate is determined to be unsuitable for entry into the study.<br>Healthy volunteers who have family members with ALS
Non-participation	110 participants were enrolled; 24 participants did not have data for at least 45 days and were therefore excluded from the analysis.
Randomization	No randomization was used.

Reporting for specific materials, systems and methods

We require information from authors about some types of materials, experimental systems and methods used in many studies. Here, indicate whether each material, system or method listed is relevant to your study. If you are not sure if a list item applies to your research, read the appropriate section before selecting a response.

Materials & experimental systems

n/a	Involved in the study	
☒	☐	Antibodies
☒	☐	Eukaryotic cell lines
☒	☐	Palaeontology and archaeology
☒	☐	Animals and other organisms
☐	☒	Human research participants
☐	☒	Clinical data
☒	☐	Dual use research of concern

Methods

n/a	Involved in the study	
☒	☐	ChIP-seq
☒	☐	Flow cytometry
☒	☐	MRI-based neuroimaging

Human research participants

Policy information about studies involving human research participants

Population characteristics	See above
Recruitment	See above
Ethics oversight	The study was approved by the institutional review board at Barrow Neurological Institute.

Note that full information on the approval of the study protocol must also be provided in the manuscript.

Clinical data

Policy information about clinical studies

All manuscripts should comply with the ICMJE guidelines for publication of clinical research and a completed CONSORT checklist must be included with all submissions.

Clinical trial registration	ClinicalTrials.gov Identifier: NCT03016897
Study protocol	Study design and data collection procedure is described in this paper: https://www.tandfonline.com/doi/pdf/10.1080/21678421.2018.1541095<br>Further details about the study are as well in: https://www.clinicaltrials.gov/ct2/show/NCT03016897?term=shefner&cond=ALS&draw=2&rank=1
Data collection	Data collection was conducted between 2017 and 2019 from participants' homes.
Outcomes	Change in Pulmonary Function [Time Frame: Daily for the first 3 months; for the subsequent 6 months measures will be obtained twice weekly]<br>Respirometer<br><br>Change in Quantitative Hand Grip [Time Frame: Daily for the first 3 months; for the subsequent 6 months measures will be obtained twice weekly]<br>Digital Handgrip Meter<br><br>Change in Electrical Impedance Myography (EIM) Measurements [Time Frame: Daily for the first 3 months; for the subsequent 6 months measures will be obtained twice weekly]<br>Skulpt Chisel<br><br>Change in Actigraphy [Time Frame: Daily for the first 3 months; for the subsequent 6 months measures will be obtained twice weekly]<br>Activity Band<br><br>ALSFRS-R [Time Frame: Weekly up to 9 months]<br>Questionnaire<br><br>Change in Patient-reported Experience measures (PREMs) [Time Frame: At Week 1, and then at 3, 6, and 9 months]<br>Questionnaire<br><br>Adverse Events [Time Frame: Monthly up to 9 months]<br>Questionnaire<br><br>Change in Voice/Speech Tracking [Time Frame: Daily for the first 3 months; for the subsequent 6 months measures will be obtained twice weekly]<br>Smartphone application
